# Trends in adrenal cancer mortality in the US: Regional, demographic and geographic disparities from 1999 to 2020

**DOI:** 10.1097/MD.0000000000045882

**Published:** 2025-11-14

**Authors:** Hassan Ijaz, Muhammad Shaheer Bin Faheem, Shamikha Cheema, Pakeeza Saif, Azka Ijaz, Shafiq Ur Rahman, Romaisa Kunwar, Syed Tawassul Hassan, Umaima Cheema, Ibrahim Manzoor, Muhammad Idrees Khan

**Affiliations:** a Department of Medicine, King Edward Medical University, Lahore, Pakistan; b Department of Medicine, Karachi Institute of Medical Sciences, KIMS, Karachi, Pakistan; c Department of Medicine, Saidu Group of Teaching Hospital Swat, Pakistan; d Department of Medicine, Karachi Medical and Dental College, KMDC, Karachi, Pakistan; e Department of Medicine, Islamic International Medical College, Riphah International University, Rawalpindi, Pakistan; f Department of Medicine, Adam University School of Medicine, Bishkek, Chuy Region, Kyrgyzstan.

**Keywords:** adrenal cancer, age-adjusted mortality rates, CDC wonder, mortality

## Abstract

Early-stage adrenal cancer has a 5-year survival rate of 50% to 60% which drops to 10% to 20% following metastasis. We aim to identify the adrenal cancer mortality patterns across various demographics and geographies in the U.S. from 1999 to 2020 by using a national database. Death certificate-associated datasets were retrieved from the CDC WONDER database using the ICD-10 code C74 to identify adrenal cancer patients. age-adjusted mortality rates were calculated per million population along with annual percent changes in them for all the stratifications, including sex, race, and geography. Overall, 14,622 mortalities were attributed to adrenal cancer, with AAMR decreasing from 2.429 in 1999 to 2.068 in 2020 (APC–0.7804; *P* <.05). Males (2.21) and non-Hispanic Whites (2.16) exhibited the highest rates. Older adults (6.09) reported the highest AAMR compared with younger populations. Further peak rates were observed among the residents of non-metropolitan areas (2.32) and the Midwest region (2.23). Overall, the mortality rates declined, but significant demographic and regional disparities exist, emphasizing the need for targeted interventions chiefly for older individuals, men, and those in non-metropolitan areas.

## 1. Introduction

Adrenal cancer is a rare but very aggressive malignancy of the adrenal cortex and presents great clinical challenges because of its poor prognosis and limited treatment options.^[1,2]^ Adrenal cancer represents only 0.02% of all cancers in the United States (U.S.), with a yearly incidence of 0.7 to 2 cases per million inhabitants, marking its rarity and diagnostic difficulty.^[3,4]^ According to surveillance, epidemiology, and end results (SEER)-based analysis, the total 5-year survival of patients with metastatic adrenal cancer remains 15% and even with advances in surgery and imaging, recent mortality patterns have continued to be worrisome.^[4^5^]^ Further, the survival rate is <1 year in those presenting with advanced stages of adrenal cancer.^[6]^ In a 2023 meta-analysis of 23 prospective trials, the median overall survival of adrenal cancer patients treated with systemic therapies such as chemotherapy, immunotherapy, or targeted therapy was between 7.4 and 11.2 months, indicating the limited effectiveness of existing treatments.^[7]^

Discrepancies in adrenal carcinoma outcomes between demographic groups have also been reported. Black and Hispanic patients have been found to have lower survival rates than non-Hispanic (NH) White patients do, even after adjustment for socioeconomic status.^[8]^ In addition, advanced age, male sex, large tumor size, and distant metastasis are known to be independent adverse prognostic factors.

Recurrence is yet another important challenge in the management of adrenal cancer. Even with complete resection and adjuvant treatments such as mitotane, disease recurrence occurs in up to 60% to 80% of patients, and the 5-year recurrence-free survival rate is as low as 33%.^[9]^ The European Network for the Study of Adrenal Tumors (ENSAT) staging system, resection margins, and response to mitotane have become significant predictors of long-term survival.^[10]^

However, recent SEER-based studies are limited in terms of geographic coverage and are focused on incidence rather than mortality, with no studies evaluating demographic and geographic mortality disparities at the national level. Thus, epidemiological investigations are needed to study adrenal cancer mortality trends and disparities across different populations. Addressing the gap, our research intends to examine trends in adrenal cancer mortality from 1999 to 2020 across various demographic and geographic subgroups, including sex, race, age, urbanization, regions, and states. This will help shape guidelines for developing targeted interventions and enhancing resource allocation to reduce adrenal cancer mortality burden, especially in high-risk populations.

## 2. Methods

### 2.1. Data source

We used the Centers for Disease Control and Prevention (CDC) wide-ranging online data for epidemiologic research (WONDER) database,^[11]^ a publicly available national mortality database constructed for epidemiologic and public health research.^[12]^ The “Multiple Cause of Death, 1999 to 2020” data were utilized to retrieve mortality statistics involving adrenal cancer in the U.S. The data included the age-adjusted mortality rates (AAMRs) of different gender and race groups in the USA up to 2020. The dataset includes data from death certificates received from all 50 states and the District of Columbia and contains information on the underlying cause of death as well as demographic variables such as age, sex, race, and ethnicity.^[12]^ For the purposes of this study, adrenal cancer-related mortality was identified through the use of the International Classification of Diseases, Tenth Revision (ICD-10) code C74, which indicates a malignant neoplasm of the adrenal gland.

### 2.2. Study population

The analysis involved patients in different age groups with adrenal cancer between 1999 and 2020 in the U.S. Cases were coded with the ICD-10 code C74 from the CDC WONDER database. Mortality data were stratified by the demographic variables of age, sex, and race/ethnicity to evaluate trends and disparities in adrenal cancer mortality in various population groups

### 2.3. Data collection and synthesis

Crude death rates and AAMRs per 1000,000 people were accessed via the CDC WONDER interface. The “Group by” option was employed to group data by demographic characteristics for comparison of groups. The Joinpoint Regression Program (version 5.4.0.0; National Cancer Institute)^[13]^ was used to calculate the annual percent changes (APCs) and average APCs in AAMR, with 95% confidence intervals (95% CI), identifying trends via Monte Carlo Permutation Tests.^[14]^ Using a 2-tailed *t*-test, APCs were categorized as ascending or descending based on whether the slope representing the change in mortality significantly differed from zero. A *P*-value < 0.05 was deemed statistically significant. This methodology facilitated the precise evaluation of adrenal cancer mortality trends over time and the identification of risk groups on the basis of demographic patterns.

### 2.4. Data privacy and confidentiality

This research utilized publicly available, deidentified, and aggregated data from the CDC WONDER database. Accordingly, no identifying personal information was accessed or contained in the analysis. Ethical approval and informed consent were unnecessary since the CDC WONDER database maintains strict data protection protocols to keep personal information private.

## 3. Results

**Figure 1.**
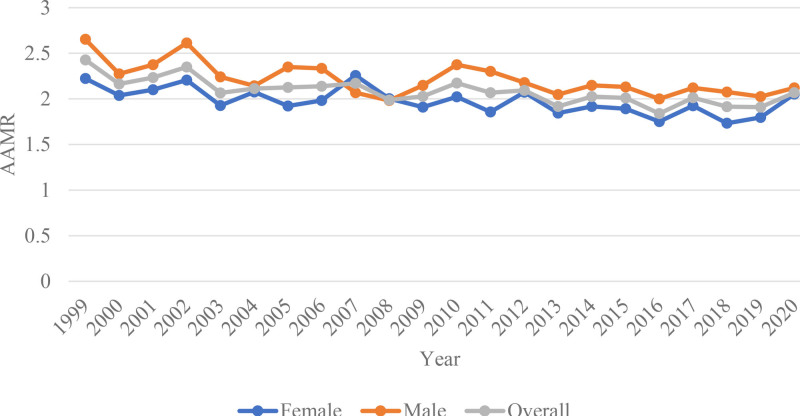
Sex-stratified and overall adrenal cancer-related AAMRs per 1000,000 people in the United States, 1999 to 2020. AAMRs = age-adjusted mortality rates.

Between 1999 and 2020, a total of 14,622 deaths were attributed to adrenal cancer. The AAMR consistently decreased, from 2.429 (95% CI: 2.245–2.614) per million in 1999 to 2.068 (95% CI: 1.918–2.219) in 2020, corresponding to an APC of–0.7804 (95% CI: −1.0804 to −0.4794, *P* = .00002). Data regarding the place of death were available for 14,533 cases, with 34.54% of deaths occurring in inpatient medical facilities, 42.75% occurring in the descendant’s home, 5.92% occurring in hospice facilities, and 9.27% occurring in nursing homes (Tables S1 and S2, Supplemental Digital Content, https://links.lww.com/MD/Q628; Fig. 1).

### 3.1. Demographic patterns

#### 3.1.1. Gender

Gender-based disparities in mortality rates were evident, with males exhibiting slightly higher mortality rates than females. The AAMR for males decreased from 2.653 (95% CI: 2.368–2.939) per million in 1999 to 2.12 (95% CI: 1.899–2.34) in 2020. A similar trend was observed in females, with the AAMR decreasing from 2.225 (95% CI: 1.983–2.467) in 1999 to 2.053 (95% CI: 1.843–2.262) in 2020. However, males experienced a more substantial decline in mortality, with an APC of–0.8453 (95% CI: −1.2479–-0.4411, *P* = .0003), compared with a gradual decrease of −0.7073 (95% CI: −1.1076–-0.3055, *P* = .0015) in females. (Tables S1 and S2, Supplemental Digital Content, https://links.lww.com/MD/Q628; Fig. 1).

#### 3.1.2. Race/ethnicity

**Figure 2.**
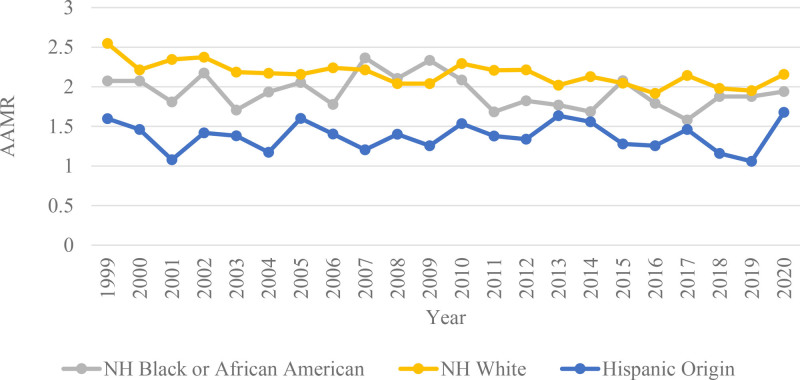
Adrenal cancer-related AAMRs per 1000,000 individuals stratified by race in the United States, 1999 to 2020. AAMRs = age-adjusted mortality rates.

Racial stratification of mortality rates revealed the highest mortality rates among the NH White population, with the AAMR decreasing from 2.548 (95% CI: 2.342–2.754) per million in 1999 to 2.157 (95% CI: 1.985–2.33) per million in 2020. This population was followed by the NH Black or African American population, where the AAMR decreased from 2.074 (95% CI: 1.59–2.659) per million in 1999 to 1.941 (95% CI: 1.55–2.397) per million in 2020. The Hispanic or Latino AAMR increased slightly from 1.598 (95% CI: 1.062–2.309) per million in 1999 to 1.677 (95% CI: 1.325–2.092) per million in 2020. The NH White population exhibited the highest APC of −0.7391 (95% CI: −1.0799–-0.3971, *P* = .0002), followed by the NH Black population, with an APC value of −0.5741 (95% CI: −1.2982–0.1552, *P* = .1159), reflecting a nonsignificant downward trend. Similarly, the Hispanic or Latino population showed a nonsignificant incline in mortality rates with an APC of 0.0158 (95% CI: −0.9697–1.0111, *P* = .9737). (Tables S1 and S2, Supplemental Digital Content, https://links.lww.com/MD/Q628; Fig. 2).

#### 3.1.3. Age

**Figure 3.**
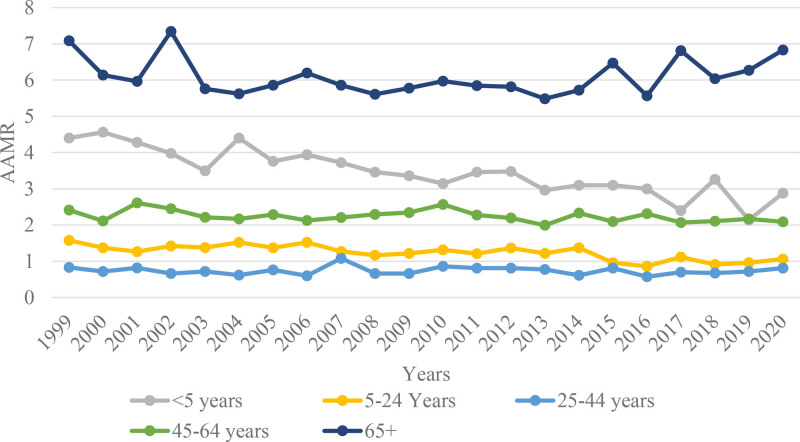
Adrenal cancer-related AAMRs per 1000,000 individuals stratified by age groups in the United States, 1999 to 2020. AAMRs = age-adjusted mortality rates.

Mortality rates were notably higher in older age groups, with the crude mortality rate (CMR) for individuals aged 65 years and above decreasing slightly from 7.08 (95% CI: 6.199–7.969) per million in 1999 to 6.83 (95% CI: 6.128–7.533) per million in 2020, reflecting an APC of 0.0057 (95% CI: −0.5722–0.5869, *P* = .9838), indicating no significant change in trend over the 2 decades. The next significant change was observed in the population under 5 years of age, where the CMR decreased from 4.4 (95% CI: 3.51–5.448) per million to 2.88 (95% CI: 2.176–3.74) per million, exhibiting the greatest decline in the CMR of any age group, with an APC of −2.4506 (95% CI: −3.0495–-1.848, *P* < .0000). For the 45 to 65 age group, the CMR decreased from 2.411 (95% CI: 2.018–2.803) per million in 1999 to 2.089 (95% CI: 1.781–2.397) per million in 2020, corresponding to an APC of −0.5149 (95% CI: −0.0553–-2.3365, *P* = .0299), which reflects a modest decrease in mortality rates over 2 decades. The CMR of the 5 to 25 age group changed from 1.576 (95% CI: 1.299–1.852) per million in 1999 to 1.059 (95% CI: 0.848–1.306) per million in 2020, showing a steady decline with an APC of −1.9302 (95% CI: −2.6674–-1.1874, *P* = .00002). The lowest mortality rates were observed in the 25 to 44-year age group, with an CMR of 0.809 (95% CI: 0.628–1.026) per million in 2020. (Tables S1 and S2, Supplemental Digital Content, https://links.lww.com/MD/Q628; Fig. 3).

#### 3.1.4. Regional patterns

**Figure 4.**
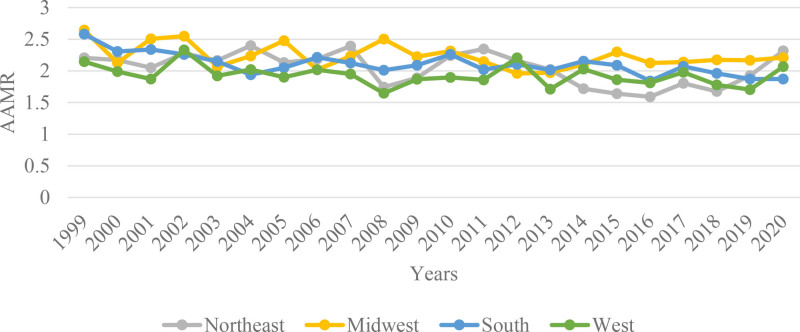
Adrenal cancer-related AAMRs per 1000,000 individuals stratified by census region in the United States, 1999 to 2020. AAMRs = age-adjusted mortality rates.

**Figure 5.**
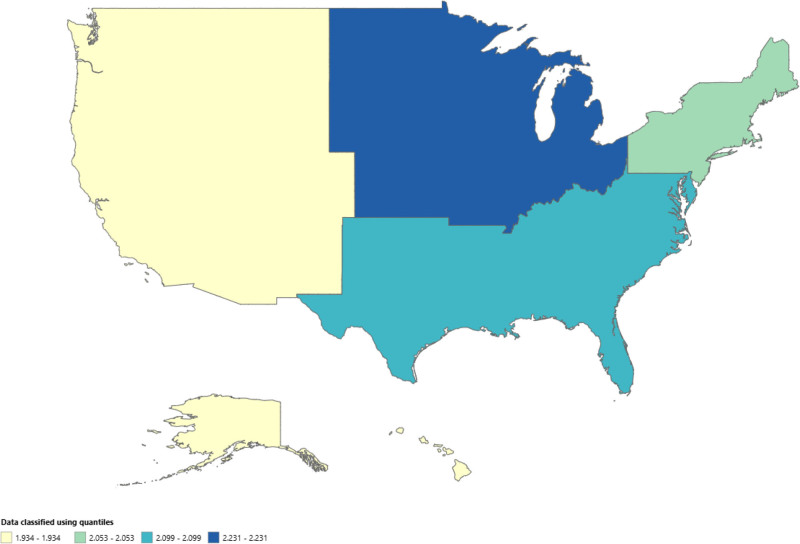
Region-level adrenal cancer-related AAMRs per 1000,000 people in the United States, 1999 to 2020. AAMRs = age-adjusted mortality rates.

Regional analysis highlighted notable differences in mortality trends across various regions. The Midwest region presented the highest mortality rates, with an AAMR decreasing from 2.645 (95% CI: 2.247–3.044) per million in 1999 to 2.211 (95% CI: 1.87–2.552) per million in 2020, reflecting an APC of −0.6321 (95% CI: −1.1500–-0.1114, *P* = .0198). The Northeast exhibited an unusual trend with a decline in mortality rates, with an AAMR of 2.205 (95% CI: 1.808–2.602) per million in 1999 to 1.676 (95% CI: 1.339–2.013) per million in 2018 at an APC value of–1.4671 (95% CI: −2.374–-0.5518, *P* = .0036). However, this was followed by a sharp increase in mortality rates, with the AAMR rising to 2.317 (95% CI: 1.926–2.708) per million in 2020, although the APC showed a nonsignificant change of 13.3068 (95% CI: −15.5583–52.039, *P* = .3825). In the South, the AAMR was 1.871 (95% CI: 1.644–2.097) per million in 2020 with an APC of −0.9340 (95% CI: −1.3157–-0.5509, *P* = .00005). The West showed a relatively modest decline in mortality, with the AAMR decreasing slightly from 2.145 (95% CI: 1.722–2.518) per million in 1999 to 2.066 (95% CI: 1.756–2.375) per million in 2020, reflecting a nonsignificant APC of −0.45 (95%CI: −1.0078–0.1175, *P* = .1140). (Tables S1 and S2, Supplemental Digital Content, https://links.lww.com/MD/Q628; Figs. 4 and 5).

#### 3.1.5. Geographic

Geographic distribution of mortality trends revealed a higher mortality burden in non-metropolitan areas. In 2020, the AAMR for non-metropolitan regions was 2.606 (95% CI: 2.161–3.052) per million in contrast to 1.971 (95% CI: 1.813–2.129) per million in metropolitan areas. Additionally, the APC exhibited a nonsignificant change in rural regions, recording a value of 0.27 (95%CI: −0.4298–0.9778, *P* = .4298), as compared to a declining trend with an APC of −0.9951 (95% CI: −1.3093–-0.6798, *P* = .0000) in metropolitan areas. (Tables S1 and S2; Fig. S1, Supplemental Digital Content, https://links.lww.com/MD/Q628).

#### 3.1.6. State

States in the top 90^th^ percentile in mortality rate were Arkansas, South Dakota, Vermont, North Dakota and Oklahoma with AAMRs at 3.018, 2.678, 2.588, 2.473 and 2.401 respectively. On the other hand, the States at the bottom 10^th^ percentile in mortality had significantly lower mortality rates and included Idaho, Hawaii, Wyoming, Utah and Connecticut with AAMRs of 1.539, 1.541, 1.621, 1.654 and 1.667 respectively. (Tables S1 and S2; Fig. S2, Supplemental Digital Content, https://links.lww.com/MD/Q628).

## 4. Discussion

We described many key findings by analyzing mortality data related to adrenal cancer extracted from the CDC WONDER database between 1999 and 2020. The analysis also revealed several important patterns in mortality rates across gender, race, place of death, U.S. census regions, and individual states. The AAMR showed a consistently decreasing trend, with most deaths occurring in inpatient medical facilities. Males presented a higher mortality rate. There were variations in the AAMR by region, with the Midwest showing the greatest value, followed by Northeast, the South, and the West. The highest AAMR was found among the NH White population, and the lowest was found in the Hispanic population. Adults over 65 years of age had the highest CMR. Arkansas, South Dakota, and Vermont were the 3 states with the highest AAMRs. Geographic analysis revealed a greater mortality burden in non-metropolitan areas. In the last 2 decades, there has been a 10-fold increase in findings related to adrenal cancer, with the increase in cross-sectional abdominal imaging studies, making it a point of interest.^[15]^

Many malignancies show sex-related disparities, largely due to the effects of sex hormones and differences in tissue differentiation between males and females.^[16,17]^ The lifetime cancer risk is 44.85% for males and 38.08% for females, with males showing a higher risk despite a shorter life expectancy.^[17]^ Our results revealed a higher mortality rate in males than in females throughout the study period, but the rate of improvement was marginally better for males than for females according to the APC. This statement contradicts the findings of Audenet et al, who reported that although most tumors occur in men, adrenal cancer, irrespective of histological classification, appears to predominantly affect females.^[18]^ Another study, which also included a pediatric population, concluded that females may have a genetic predisposition to adrenal carcinoma. However, this finding has also been disputed.^[2]^ These sex disparities are influenced by sex-specific risk factors, such as higher smoking rates in men and the use of oral contraceptives in females, especially at a young age.^[2,19]^ Sex-specific differences significantly influence the presentation, genetic mutations, and clinical outcomes of benign adrenal tumors, underscoring the need for sex-tailored diagnostic and treatment strategies.^[20]^ There is a need to investigate the underlying factors contributing to the higher male-associated mortality rate from adrenal cancer.

Geographic disparities were also studied in this pooled analysis. The Midwest region presented the highest mortality rate among all U.S. regions, with only a subtle decline observed over the past 2 decades. Furthermore, our study revealed that rural and non-metropolitan areas contributed more significantly to mortality rates in the U.S. This starkly underscores the profound and unacceptable failure to provide adequate healthcare access to the rural population. Rural–urban disparities in cancer incidence and mortality are partly due to limited access to healthcare in rural areas. Factors include financial barriers such as lack of insurance, a shortage of physicians, and a significant deficit in oncologist availability. However, the urban population has more than 3 times more specialists per capita than the rural population does, affecting access to cancer screening and treatment. Many rural counties lack oncologists, further impeding early detection.^[21]^

Our study revealed the lowest mortality rates among individuals aged 25 to 44 years, which is consistent with a Minnesota-based study that reported increased adrenal cancer incidence beginning at age 40.^[15]^ This finding aligns with our finding that the highest mortality rates occur in individuals aged 65 and above. Many general factors contribute to disease in elderly individuals, such as decreased mobility, malnourishment, immunosenescence and polypharmacy.^[22]^ Our study aligns with that of Constantine et al, who reported a bimodal age distribution, with the first peak occurring before age 5 and the second occurring during the fourth to fifth decades of life^,[23]^ but it can occur at all ages.^[24]^

The NH White population experienced the highest burden of this cancer, followed by the NH Black population, whereas Hispanics had the lowest incidence. This aligns with findings that African Americans were not associated with higher disease stage at diagnosis or poorer survival outcomes^,[25]^ whereas NH White Americans dominated.^[26]^ However, a 4-year study revealed that minority patients, who composed 22.6% of the cohort (69.9% NH White), experienced higher complication rates and longer hospital stays after adrenalectomy, partly due to reduced access to high-volume surgeons despite similar comorbidities.^[27]^ Conversely, another study reported no significant differences in survival outcomes by race.^[28]^ These results could also be because of the underestimation observed among NH Black and Hispanic individuals compared with NH White individuals due to conscious or unconscious biases, including medical decision-making bias, test bias, or survival bias.^[29]^ Additionally, Hispanic individuals tend to be diagnosed at a younger age than Caucasians are, although this earlier onset is not associated with a more advanced tumor stage or poorer prognosis.^[30]^

Our research highlights the effectiveness of current treatments for adrenal carcinoma; however, improvements in survival rates remain uneven, with older adults showing comparatively smaller gains. Given the persistent disparities across genders and geographic regions, healthcare professionals should focus on enhancing access to care and implementing targeted, community-specific intervention strategies.

One limitation of our study is its retrospective design, which relies on secondary data and restricts our ability to establish direct associations between treatments and observed mortality rates. Additionally, the use of ICD codes and death certificates may lead to potential misclassification or misdiagnosis, coding errors, and underreporting of adrenal carcinoma-related mortality. Lastly, as the database includes only U.S. citizens, deaths among nonresidents are excluded.

## 5. Conclusions

Our study reported a modest decline in adrenal cancer mortality between 1999 and 2020 and revealed significant disparities that exist in various demographic and geographic populations. This comprehensive national analysis of mortality trends across sex, age, race/ethnicity and geography extends beyond the SEER-based incidence data by providing a detailed assessment of populations at risk while emphasizing the need to develop targeted interventions and allocation of resources to address these inequalities.

## Acknowledgments

This work was initially presented in the form of an abstract at the 2025 ASCO Annual Meeting (Journal of Clinical Oncology, 2025; 43:16_suppl. e23069). However, the current manuscript significantly expands on the abstract, providing detailed methodology and a comprehensive analysis of demographic and geographic subgroups, including state-level, metropolitan, and non-metropolitan comparisons. Further, it provides an in-depth discussion of the observed disparities.

## Author contributions

**Conceptualization:** Hassan Ijaz, Shamikha Cheema.

**Data curation:** Hassan Ijaz, Shamikha Cheema, Pakeeza Saif.

**Formal analysis:** Hassan Ijaz, Shamikha Cheema, Pakeeza Saif.

**Investigation:** Muhammad Shaheer Bin Faheem, Shafiq Ur Rahman.

**Methodology:** Muhammad Shaheer Bin Faheem, Shafiq Ur Rahman.

**Project administration:** Hassan Ijaz, Muhammad Shaheer Bin Faheem, Shafiq Ur Rahman, Syed Tawassul Hassan, Muhammad Idrees Khan.

**Resources:** Muhammad Shaheer Bin Faheem, Azka Ijaz, Shafiq Ur Rahman, Umaima Cheema, Muhammad Idrees Khan.

**Software:** Pakeeza Saif, Azka Ijaz.

**Supervision:** Hassan Ijaz.

**Validation:** Shamikha Cheema, Azka Ijaz, Ibrahim Manzoor.

**Visualization:** Pakeeza Saif, Azka Ijaz, Romaisa Kunwar.

**Writing – original draft:** Hassan Ijaz, Shamikha Cheema, Pakeeza Saif, Azka Ijaz, Romaisa Kunwar, Syed Tawassul Hassan, Ibrahim Manzoor, Umaima Cheema.

**Writing – review & editing:** Hassan Ijaz, Shamikha Cheema, Pakeeza Saif, Azka Ijaz, Romaisa Kunwar, Syed Tawassul Hassan, Ibrahim Manzoor, Umaima Cheema.

## Supplementary Material


