# Supplementary material for: Trends in adrenal cancer mortality in the US: Regional, demographic and geographic disparities from 1999 to 2020
Source: Medicine (Baltimore). 2025 Nov 14;104(46):e45882. doi: 10.1097/MD.0000000000045882 (PMC12622733; doi:10.1097/MD.0000000000045882)
Supplement: Supplementary file 1 [file medi-104-e45882-s001.docx]

Supplemental Table 1. Total deaths and average age-adjusted mortality rates for adrenal cancer by demographic, regional, and geographic characteristics in the United States, 1999–2020.

| **Variable** | **Adrenal Cancer related Deaths (n)** | **Age Adjusted Mortality Rate (AAMR) per 1,000,000 ^a^** |
| --- | --- | --- |
| Overall Population | 14,622 | 2.09 (2.06 – 2.13) |
| Sex |  |  |
| Male  Female | 7,270  7,352 | 2.21 (1.97 – 2.45)  1.97 (1.76 – 2.19) |
| US Census Region |  |  |
| Northeast  Midwest  South  West | 2,622  3,470  5,459  3,071 | 2.04 (1.67 – 2.42)  2.23 (1.88 – 2.58)  2.10 (1.84 – 2.37)  1.93 (1.61 – 2.26) |
| Race/Ethnicity |  |  |
| Hispanic  NH Black or African American  NH White | 1,133  1,671  12,473 | 1.37 (0.99 – 1.85)  1.93 (1.51 – 2.44)  2.16 (1.98 – 2.34) |
| Age |  | **Crude Mortality Rate (CMR) per 1,000,000** |
| <5 years  5-25 years  25-44 years  45-64 years  65+ years | 1,511  2,220  1,349  3,934  5,608 | 3.46 (2.70 – 4.38)  1.24 (1.01 – 1.50)  0.74 (0.56 – 0.95)  2.24 (1.91 – 2.58)  6.09 (5.33 – 6.84) |
| **Place of Death** ^c^ |  | **% of Total Deaths** |
| Medical facilities-Inpatient  Medical facility-Outpatient or ER  Hospice Facility  Decedent’s Home  Nursing/Long Term Care  Others | 5,051  367  866  6,253  1,356  640 | 35  3  6  43  9  4 |
| **Urbanization** |  |  |
| Urban  Rural | 11,971  2,651 | 2.038 (1.87 – 2.21)  2.32 (1.89 – 2.74) |

**Note:**

^a^ Values represent the average AAMR across the entire study period (1999–2020). AAMRs are reported per 1,000,000 population with 95% confidence intervals. Case numbers reflect total deaths during the study period.

^b^ Age-adjusted mortality rate (AAMR) is not applicable for place of death.

Supplemental Table 2. Annual percentage changes (APCs) and average annual percentage changes (AAPCs) in adrenal cancer mortality in the USA from 1999 to 2020.

| **Variable** | **AAPC (95% CI)** | **Trend segment** | **Year** | **APC (95% CI)** |
| --- | --- | --- | --- | --- |
| Overall population | -0.7804* (-1.0804 to -0.4794) | 1 | 1999-2020 | -0.7804* (-1.0804 to -0.4794) |
| **Sex** |  |  |  |  |
| Female  Male | -0.7073* (-0.7073 to -1.1076)  -0.8453* (-1.2479 to -0.4411) | 1  1 | 1999-2020  1999-2020 | -0.7073* (-1.1076 to -0.7073)  -0.8453* (-1.2479 to -0.4411) |
| **US Census Region** |  |  |  |  |
| Northeast  Midwest  South  West | -0.1473 (-2.8219 to 2.6009)  -0.6321* (-1.1500 to -0.1114)  -0.9340* (-1.3157 to -0.5509)  -0.4467 (-1.0078 to 0.1175) | 1  2  1  1  1 | 1999-2018  1999-2020  1999-2020  1999-2020  1999-2020 | -1.4671* (-2.3740 to -0.5518)  13.3068 (-15.558 to 52.0390)  -0.6321* (-1.1500 to -0.1114)  -0.9340* (-1.3157 to -0.5509)  -0.4467 (-1.0078 to 0.1175) |
| **Race/Ethnicity** |  |  |  |  |
| Hispanic  NH Black  NH White | 0.0158 (-0.9697 to 1.0111)  -0.5741 (-1.2982 to 0.1552)  -0.7391* (-1.0799 to -0.3971) | 1  1  1 | 1999-2020  1999-2020  1999-2020 | 0.0158 (-0.9697 to 1.0111)  -0.5741 (-1.2982 to 0.1552)  -0.7391* (-1.0799 to -0.3971) |
| **Urbanization** |  |  |  |  |
| Urban  Rural | -0.9951*(-1.3093 to -0.6798)  0.2715 (-0.4298 to 0.9778) | 1  1 | 1999-2020  1999-2020 | -0.9951*(-1.3093 to -0.6798)  0.2715 (-0.4298 to 0.9778) |
| **Age** |  |  |  |  |
| <5 year  5-24 years  25-44 years  45-64 years  65+ years | -2.4506* (-3.0495 to -1.848)  -1.9302* (-2.6674 to -1.1874)  -0.2254 (-1.3075 to 0.8686)  -0.5149* (-0.9724 to -0.0553)  0.0057 (-0.5722 to 0.5869) | 1  1  1  1  1 | 1999-2020  1999-2020  1999-2020  1999-2020  1999-2020 | -2.4506* (-3.0495 to -1.848)  -1.9302* (-2.6674 to -1.1874)  -0.2254 (-1.3075 to 0.8686)  -0.5149* (-0.9724 to -0.0553)  0.0057 (-0.5722 to 0.5869) |

Note:

- *Annual percentage change (APC) is significantly different from zero at the alpha = 0.05 level.
- AAPC represents the average annual percent change across the entire study period (1999–2020). APC values represent segment-specific annual percent changes identified by Joinpoint regression, and multiple segments may be present for some groups

Supplementary Figure 1: Adrenal cancer-related age-adjusted mortality rates (AAMRs) per 1,000,000 individuals stratified by urbanization in the United States, 1999 to 2020.


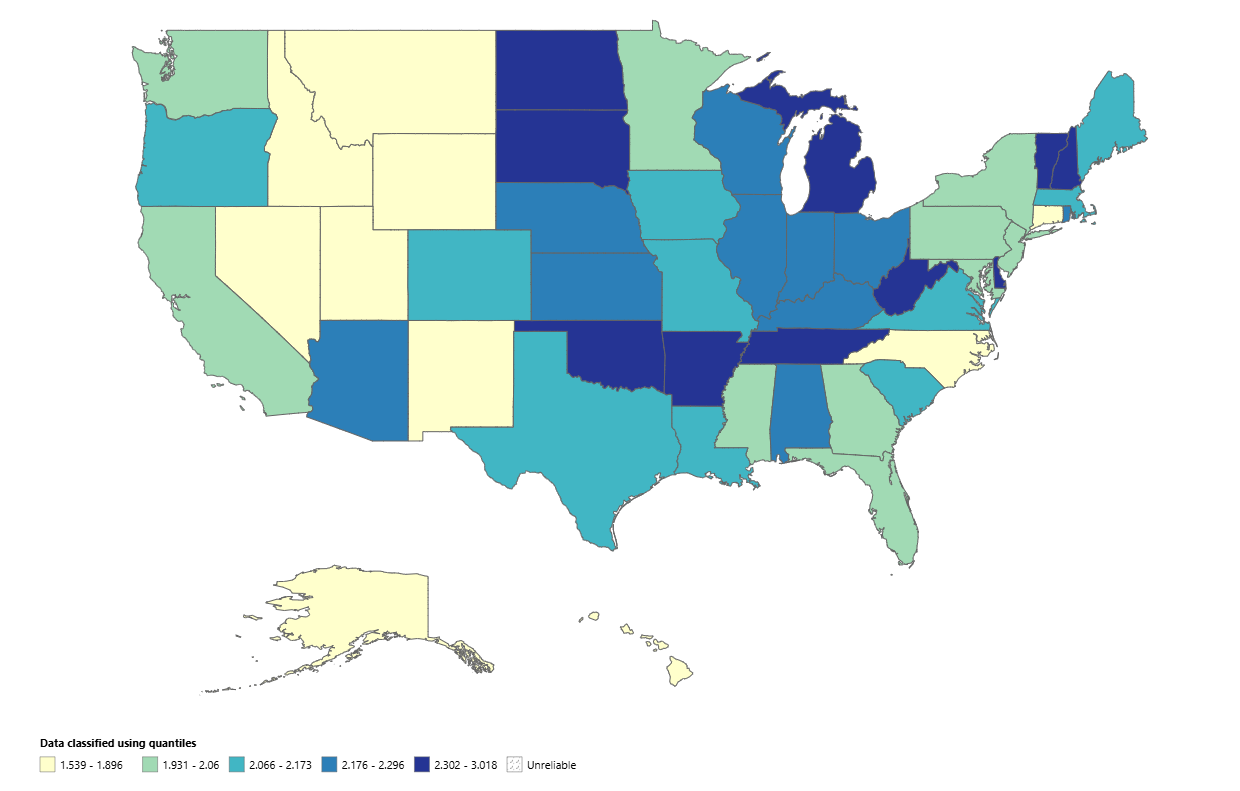


Supplementary Figure 2: State-level adrenal cancer-related age-adjusted mortality rates (AAMRs) per 1,000,000 people in the United States, 1999 to 2020.
